# Can Hantavirus cruise into the next pandemic?

**DOI:** 10.1590/0074-02760260231

**Published:** 2026-10-19

**Authors:** Nias YG Peng, Alejandro Rojas-Fernandez, Joshua M Deerain, Julio Aguado, Marcio RT Nunes, Alberto A Amarilla

**Affiliations:** 1 No current affiliation - Previously Commonwealth Scientific and Industrial Research Organization, Health & Biosecurity, Black Mountain, Canberra, Australia Commonwealth Scientific and Industrial Research Organization Health & Biosecurity Canberra Australia; 2 Universidad Austral de Chile, Faculty of Medicine, Institute of Medicine, Valdivia, Chile Universidad Austral de Chile Faculty of Medicine Institute of Medicine Valdivia Chile; 3 Commonwealth Scientific and Industrial Research Organization, Australian Centre for Disease Preparedness, East Geelong, Australia Commonwealth Scientific and Industrial Research Organization Australian Centre for Disease Preparedness East Geelong Australia; 4 University of Colorado, Department of Pharmaceutical Sciences, Aurora, Colorado, USA University of Colorado Department of Pharmaceutical Sciences Aurora Colorado United States; 5 Universidade Federal do Pará, Instituto de Ciências Biológicas, Laboratório de Tecnologia Biomolecular, Belém, PA, Brasil Universidade Federal do Pará Instituto de Ciências Biológicas Laboratório de Tecnologia Biomolecular Belém PA Brazil

**Keywords:** Hantavirus, Andes virus, M/V *Hondius*

## Abstract

Despite concerns arising from a recent ship-associated outbreak on the *M/V Hondius* cruise, current evidence suggests that Andes virus (ANDV) poses a low pandemic risk. Nevertheless, viral evolution, ecological changes, and reservoir host dynamics could drive future emergence of ANDV, underscoring the need for improved surveillance, diagnostics, therapeutics, and vaccine preparedness.

Hantaviruses are part of the family *Hantaviridae*, which comprises eight genera.^1^ Among them, viruses associated with human disease belong to the genus *Orthohantavirus*.^2^,^3^ Orthohantaviruses are enveloped, tri-segmented, negative-sense RNA viruses whose genome consists of small (S), medium (M), and large (L) segments encoding the nucleocapsid protein (N), glycoproteins Gn/Gc, and the RNA-dependent RNA polymerase (RdRp), respectively.^4^,^5^,^6^

The two major clinical diseases caused by orthohantaviruses are haemorrhagic fever with renal syndrome (HFRS) and hantavirus pulmonary syndrome/cardiopulmonary syndrome (HPS/HCPS).^3^,^7^ HFRS is mainly caused by Asian and European orthohantaviruses, including Hantaan virus, Seoul virus, Puumala virus, and Dobrava virus, and primarily affects the kidneys.^2^ In contrast, HPS/HCPS is caused by several American orthohantaviruses, including *Orthohantavirus sinnonbrenense* (Sin Nombre virus; SNV) and *Orthohantavirus andesense* (Andes virus; ANDV), and is characterised by severe respiratory disease, pulmonary oedema, and cardiovascular compromise.^2^ Importantly, ANDV-associated HPS is characterised by high case-fatality rates, with mortality estimates in Chile and Argentina ranging from approximately 21% to 50%.^8^,^9^,^10^,^11^,^12^

Orthohantavirus infection is primarily acquired by inhaling aerosolised particles contaminated with the urine, saliva, or faeces of infected rodents, although transmission may also occur through direct contact with contaminated materials or rodent bites.^2^,^3^ While most orthohantaviruses are transmitted exclusively from rodents to humans, ANDV is unique among orthohantaviruses for its demonstrated capacity for person-to-person transmission, particularly during close, prolonged contact with symptomatic individuals.^9^,^12^,^13^,^14^,^15^ Although sustained community transmission has not been demonstrated, ANDV remains the only orthohantavirus for which person-to-person transmission has been consistently documented, predominantly among close household or intimate contacts. On 4 May 2026, an international cluster of ANDV infections was identified among passengers travelling aboard the *M/V Hondius* following its departure from Ushuaia, Argentina. The event prompted a multinational public health response involving case isolation, quarantine, surveillance, contact tracing, and genomic investigation.^16^ The WHO declared the outbreak over on 2 July 2026, with 13 cases reported, including 3 fatalities.^17^ The *M/V Hondius* cluster therefore provides an important contemporary example of ANDV’s epidemic potential.

## Could ANDV cause a future pandemic?

Despite documented person-to-person transmission associated with the *M/V Hondius* cluster, current epidemiological and genomic evidence indicates that the likelihood of this outbreak progressing to sustained epidemic or pandemic transmission was exceedingly low. Prompt implementation of public health interventions, including quarantine measures and active surveillance of individuals aboard the vessel, facilitated the early identification and isolation of suspected cases. Furthermore, genomic analyses revealed remarkably limited viral diversity among sequenced cases from the *M/V Hondius* cluster, with identical S and M segment sequences and only two synonymous single-nucleotide polymorphisms (SNPs) detected within the L segment, one in case 5 and another in case 7. Importantly, no evidence of amino acid changes associated with enhanced transmissibility or sustained human-to-human spread was identified.^18^ In contrast, the outbreak sequences showed approximately 98.7% nucleotide identity across all three genome segments with ANDV sequences identified in Argentina in 1997 and 2018, indicating considerably greater divergence from these historical reference sequences.^18^ Furthermore, the reported positive cases fell within the incubation period of nine to 45 days for ANDV, suggesting that the cluster may have originated from a limited zoonotic exposure event, potentially followed by restricted secondary transmission, and is unlikely to develop into an epidemic or a pandemic event. However, given that ANDV has historically caused multiple outbreaks, it is important to highlight the conditions that could enable localised ANDV outbreaks to evolve and escalate into larger epidemics, and potentially, under exceptional circumstances, into a pandemic threat among human populations, as illustrated in Figure.

**Figure:**
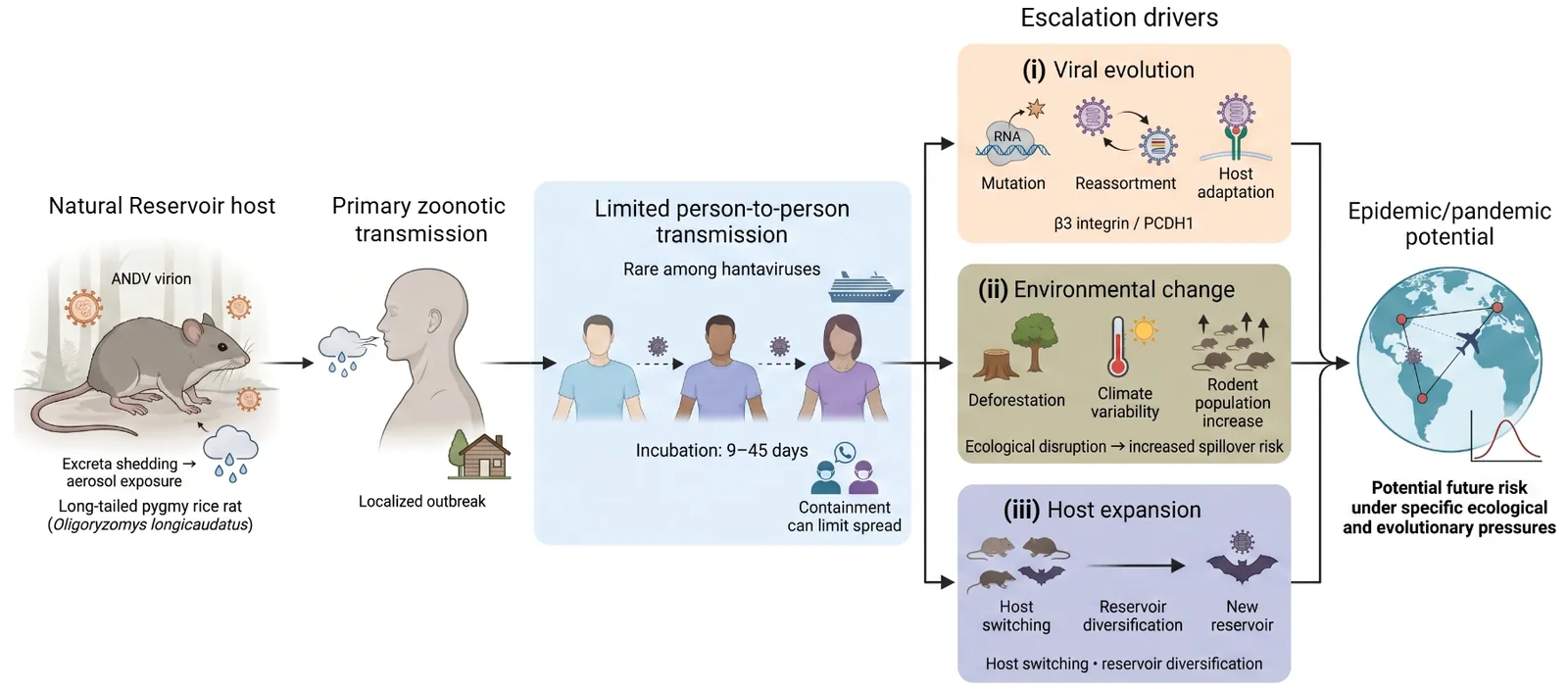
Potential drivers of Andes virus (ANDV) outbreak escalation and pandemic emergence. ANDV is maintained in rodent reservoirs, primarily the long-tailed pygmy rice rat (*Oligoryzomys longicaudatus*), and is transmitted to humans through inhalation of aerosolised rodent excreta. Unlike other orthohantaviruses, ANDV can also undergo limited person-to-person transmission, although sustained community transmission has not been documented. The graphical abstract highlights three factors that could contribute to outbreak escalation: (i) viral evolution through mutation, reassortment, and host adaptation; (ii) environmental and ecological changes that increase rodent abundance and spillover risk; and (iii) expansion of reservoir host range through host switching and reservoir diversification. Under favourable ecological and evolutionary conditions, these processes could increase the scale, frequency, geographic distribution, or transmission potential of future outbreaks, underscoring the importance of continued surveillance, preparedness, and the development of effective diagnostics, therapeutics, and vaccines. Illustration created with BioRender.com.

*First* - The acquisition of beneficial mutations may facilitate adaptation to human hosts. Genetic variation arising during viral replication could theoretically influence viral fitness, host range, or transmission efficiency under selective pressures. Orthohantaviruses replicate using an error-prone RNA-dependent RNA polymerase (RdRp), with evolutionary rates estimated at approximately 10⁻²-10⁻⁴ substitutions per site per year, consistent with rates reported for other RNA viruses.^19^ However, the relationship between genomic variation and altered transmissibility in ANDV remains incompletely understood, and no specific adaptive mutations have yet been conclusively associated with sustained human-to-human transmission.

Previous studies have shown that ANDV strains from rodent and human hosts can produce markedly different outcomes in the Syrian hamster model.^20^,^21^ Notably, a Chilean human isolate (CHI-7913), recovered from a fatal HPS case, acquired only a single non-synonymous mutation after 25 serial passages in hamsters and remained non-pathogenic, indicating relative genomic stability.^20^ In contrast, the rodent-derived isolate Chile-9717869 causes uniformly lethal disease despite differing by 23 amino acids from CHI-7913.^21^ Although these findings suggest a degree of genomic stability, they do not necessarily preclude adaptation. One possible explanation is that the CHI-7913 isolate had already undergone host-specific adaptation following zoonotic transmission to humans, thereby enhancing fitness in human cells while reducing replication efficiency and pathogenicity in the Syrian hamster model. Collectively, these observations indicate that relatively few genetic changes can substantially alter host adaptation and disease phenotype in ANDV, while the molecular determinants governing virulence and transmissibility remain incompletely understood.

Several biological mechanisms may contribute to these processes, including alterations in receptor usage, interferon antagonism, and genome reassortment. Pathogenic orthohantaviruses utilise β_3_ integrins, protocadherin-1, and other endothelium-associated receptors for host-cell entry, and mutations affecting glycoprotein-receptor interactions could alter host permissiveness and enhance adaptation to humans.^22^,^23^,^24^,^25^,^26^ In addition, the ability of orthohantaviruses to suppress interferon signalling contributes to reservoir persistence and may influence pathogenicity across different hosts.^27^,^28^,^29^,^30^,^31^ Importantly, the segmented orthohantavirus genome permits genetic reassortment during co-infection, potentially generating novel phenotypic combinations with altered virulence, transmissibility, or host range.^32^ Although reassortment appears less frequent than in other members of the order *Bunyavirales* (*e.g.* orthobunyaviruses) and influenza viruses, it remains an important mechanism contributing to long-term evolutionary adaptation and host expansion.^33^

*Second* - An increase in rodent reservoir populations driven by environmental and ecological changes may amplify the risk of zoonotic spillover. Environmental disruption is a well-recognised determinant of orthohantavirus emergence and transmission dynamics.^34^,^35^ Climatic oscillations, including El Niño-associated rainfall patterns, can substantially increase rodent abundance and population density, thereby elevating opportunities for human exposure, as previously documented during Sin Nombre virus (SNV) outbreaks in North America. In South America, deforestation, agricultural expansion, urban encroachment into rural and forested ecosystems, and biodiversity disturbance may similarly intensify contact between humans and ANDV reservoir hosts. In parallel, globalisation and expanding transportation networks have facilitated the international dissemination of rodent-associated orthohantaviruses, as illustrated by the spread of Seoul virus (SEOV) through maritime trade and the pet rat industry.^36^ Collectively, these ecological and anthropogenic pressures may increase the frequency of spillover events and the geographic distribution of orthohantavirus exposure. However, there is currently no evidence that such conditions alone are sufficient to drive sustained human-to-human transmission or pandemic-level spread of ANDV.

*Third* - Expansion of reservoir host range may contribute to broader geographic dissemination and increased opportunities for human exposure. Orthohantaviruses were historically considered highly host-specific pathogens, with individual virus lineages predominantly associated with a single rodent reservoir species through long-term co-evolutionary relationships.^37^,^38^ ANDV is primarily associated with *Oligoryzomys longicaudatus* as its principal reservoir host. However, accumulating phylogenetic and ecological evidence has challenged the concept of strict host specificity, demonstrating that host-switching events, spillover infections, and reservoir diversification have likely occurred repeatedly throughout orthohantavirus evolutionary history.^19^,^39^,^40^ In addition, the identification of genetically divergent orthohantaviruses in shrews, moles, and bats suggests that ancestral orthohantaviruses may have undergone multiple historical cross-species transmission events.^39^,^41^,^42^ Collectively, these findings indicate that orthohantavirus host associations, although generally stable, are not biologically absolute. Habitat disruptions, altered species interactions, and environmental pressures could therefore increase opportunities for cross-species transmission and geographic expansion over extended ecological timescales, potentially increasing the frequency of human exposure to infected reservoir hosts. Nevertheless, there is currently no evidence that such ecological processes have resulted in ANDV lineages with enhanced sustained human-to-human transmissibility.

## Current preparedness against ANDV outbreaks

Perhaps more worrying is the limited availability of rapid diagnostics, antivirals, and vaccines for responding to outbreaks of ANDV and other clinically relevant orthohantaviruses. Several antiviral and immunotherapeutic strategies have been explored against human disease-causing orthohantaviruses, including nucleoside analogues such as ribavirin, favipiravir and ETAR; entry inhibitors such as lactoferrin and griffithsin; host-directed approaches targeting vascular leakage pathways; RNAi-based strategies; and, more recently, neutralising monoclonal antibodies targeting the Gn/Gc glycoprotein complex.^(2,3,43-48)^ However, no orthohantavirus-specific antiviral has yet become an established standard of care, and most candidates remain experimental or dependent on very early administration. In parallel, vaccine development efforts have advanced substantially over the past decades. Hantavax^®^, an inactivated orthohantavirus vaccine marketed in South Korea to prevent HFRS caused by Hantaan and Seoul viruses, remains among the only orthohantavirus vaccines licensed for human use, while several next-generation vaccine candidates are currently under development and evaluation in preclinical and early-phase clinical trials.^43^,^49^ Despite these advances, effective countermeasures specifically targeting ANDV remain limited, highlighting an important preparedness gap for future outbreaks.

*In conclusion* - Overall, current evidence does not support the conclusion that ANDV is imminently poised to emerge as a pandemic pathogen comparable to severe acute respiratory syndrome coronavirus 2 (SARS-CoV-2). Nevertheless, under favourable ecological and epidemiological conditions, localised ANDV outbreaks may increase in scale or geographic distribution, potentially posing broader regional public health challenges. These considerations underscore the importance of strengthening preparedness strategies to prevent the escalation of orthohantavirus outbreaks, particularly in under-resourced endemic regions of South America. Integrated ecological surveillance approaches, including climate-informed forecasting of rodent population dynamics,^50^ together with genomic epidemiological surveillance of ANDV and other clinically relevant orthohantaviruses, may improve early detection of spillover risk, characterise orthohantavirus diversity and evolutionary dynamics, and support timely public health responses. In addition, expanded availability of antiviral therapeutics, monoclonal antibodies, preventive vaccines, and rapid diagnostic platforms will be important for comprehensive orthohantavirus outbreak preparedness and response. These diagnostic platforms could include lateral-flow assays to detect orthohantavirus-specific IgM and IgG antibodies in symptomatic patients,^51^ as well as antigen-based tests for direct virus detection in both humans and reservoir hosts.

## Data Availability

The contents underlying the research text are included in the manuscript.
