# Supplementary material for: Can Hantavirus cruise into the next pandemic?
Source: Mem Inst Oswaldo Cruz. 2026 Oct 19;121:e260231. doi: 10.1590/0074-02760260231 (PMC13679879; doi:10.1590/0074-02760260231)
Supplement: Peer review history

# Reviewer report: FIRST REVIEW ROUND - REVIEWERS’ COMMENTS

## REVIEWER #1

The manuscript titled “Could Hantavirus Transmit the Next Pandemic?” presents a compelling and relevant discussion regarding the potential for hantavirus cardiopulmonary syndrome (HCPS) outbreaks. Prompted by the recent atypical HCPS outbreak associated with Andes orthohantavirus (ANDV) aboard the cruise ship M/V Hondius, the authors analyze this threat from virological, genomic, and eco-epidemiological perspectives. They convincingly argue that sustained person-to-person transmission would require highly specific—and currently unlikely—evolutionary conditions.

The manuscript, from the abstract to the main text and references, is clear and well-structured. Notably, the authors present robust evidence that ANDV currently lacks the adaptive mutations necessary to drive person-to-person transmission in the manner observed with influenza viruses or SARS-CoV-2. This provides a timely and necessary clarification: although hantaviruses possess a trisegmented RNA genome capable of rapid mutation, reassortment and host adaptation, a large-scale epidemic remains highly unlikely. Finally, by advocating for an integrated global approach—combining genomic-epidemiological monitoring, rapid diagnostics, and accessible therapies—the authors highlight the precise surveillance strategies needed to contain localized HCPS outbreaks before they escalate into regional public health threats. Thus, in addition to adequately answering the title’s central question, the authors highlight the urgent global need for continuous, targeted surveillance—adopting a One Health approach—in areas endemic for SCPH, as well as for the accelerated development of effective diagnostics, specific therapies, and vaccines against HCPS.

Minor comments: (i) standardized taxonomy — replace the word “hantavirus” with “orthohantavirus”, and (ii) in the graphical abstract : Oligoryzomys longicaudatus has been correctly italicized and change “exposure to aerosolised” to “inhalation of aerosolized.”

## REVIEWER #2

This manuscript presents an interesting ecoevolutionary perspective into the outstanding question of Hantavirus’ pandemic potential. It has been discussed long before the Hondius outbreak that hantaviruses are worth being surveilled and studied to better understand if through evolution and expansion of host distributions and rodent-human contact interfaces, Hantaviruses could become the agents behind the next pandemic. The authors create a solid compendium of evidence regarding these scenarios and provide a grounded commentary on ANDV’s lack of pandemic potential at the moment, bringing a sobering message at a time when the lay public needs a better understanding of Hantaviruses.

That being said, I would like to suggest some minor changes to enhance the flow of the manuscript:

1. In the background section, there should be 2-3 sentences introducing the reader to the M/V Hondius situation. At the time of this review, the Hondius is not at the front page of the news anymore, and moreover, the manuscript should be timeless. For that reason, it would be good if the authors could include a section explaining the Hondius outbreak.

2. Related to the first point, in lines 64-66, the authors dive right into discussing the “likelihood that the M/V Hondius ANDV cluster could progress into a sustained epidemic”. Before this section, there is no context of what the N/V Hondius ANDV cluster is.

3. In lines 69-70, the authors mention that the sequences derived from the ANDV cluster have two synonymous mutations with respect to ANDV reference sequences from Argentina (1997 and 2018) and cite the Virological.org post by Dr. Gustavo Palacios. However, that is not what the post mentions; instead, the post describes two nonsynonymous mutations arising in the L segment between the first three sequences generated in South Africa, and they argue that this is a sign of a single spillover event as the origin of the index case. The original publication then mentions “98.76% and 98.75% based on the L segment, 98.71% and 98.68% on the M segment and 98.73% and 98.73% on the S segment”, these are with respect to the reference sequences from 1997 and 2018, which is more than two SNPs. Therefore, the authors should rectify the assertion of only two SNPs in the Hondius sequences, it would be evolutionarily impossible for an RNA virus to only accumulate two mutations in almost 30 years.

## AUTHORS’ RESPONSE TO THE REVIEWERS

Response to Reviewers

## Reviewer 1:

The manuscript titled “Could Hantavirus Transmit the Next Pandemic?” presents a compelling and relevant discussion regarding the potential for hantavirus cardiopulmonary syndrome (HCPS) outbreaks. Prompted by the recent atypical HCPS outbreak associated with Andes orthohantavirus (ANDV) aboard the cruise ship M/V Hondius, the authors analyze this threat from virological, genomic, and eco-epidemiological perspectives. They convincingly argue that sustained person-to-person transmission would require highly specific—and currently unlikely—evolutionary conditions.

The manuscript, from the abstract to the main text and references, is clear and well-structured. Notably, the authors present robust evidence that ANDV currently lacks the adaptive mutations necessary to drive person-to-person transmission in the manner observed with influenza viruses or SARS-CoV-2. This provides a timely and necessary clarification: although hantaviruses possess a trisegmented RNA genome capable of rapid mutation, reassortment and host adaptation, a large-scale epidemic remains highly unlikely. Finally, by advocating for an integrated global approach—combining genomic-epidemiological monitoring, rapid diagnostics, and accessible therapies—the authors highlight the precise surveillance strategies needed to contain localized HCPS outbreaks before they escalate into regional public health threats. Thus, in addition to adequately answering the title’s central question, the authors highlight the urgent global need for continuous, targeted surveillance—adopting a One Health approach—in areas endemic for SCPH, as well as for the accelerated development of effective diagnostics, specific therapies, and vaccines against HCPS.

Minor comments: (i) standardized taxonomy — replace the word “hantavirus” with “orthohantavirus”, and (ii) in the graphical abstract: Oligoryzomys longicaudatus has been correctly italicized and change “exposure to aerosolised” to “inhalation of aerosolized.”

Authors’ response:

Thank you for reviewing our manuscript and providing us with useful feedback. We have addressed your comments accordingly.

We have standardized the taxonomy by replacing the word “hantavirus” with “orthohantavirus” found on lines 43, 45, 51, 54, 55, 58, 84, 104, 108, 109, 117, 125, 127, 131, 138, 139, 141, 151, 152, 159, 160, 163, 172, 175, 176, 179, 182, 189, 206.

We have made the recommended changes in the graphical abstract figure legend from “exposure to aerosolised” to “inhalation of aerosolised” on Line 206.

## Reviewer 2:

This manuscript presents an interesting ecoevolutionary perspective into the outstanding question of Hantavirus’ pandemic potential. It has been discussed long before the Hondius outbreak that hantaviruses are worth being surveilled and studied to better understand if through evolution and expansion of host distributions and rodent-human contact interfaces, Hantaviruses could become the agents behind the next pandemic. The authors create a solid compendium of evidence regarding these scenarios and provide a grounded commentary on ANDV’s lack of pandemic potential at the moment, bringing a sobering message at a time when the lay public needs a better understanding of Hantaviruses.

That being said, I would like to suggest some minor changes to enhance the flow of the manuscript:

1. In the background section, there should be 2-3 sentences introducing the reader to the M/V Hondius situation. At the time of this review, the Hondius is not at the front page of the news anymore, and moreover, the manuscript should be timeless. For that reason, it would be good if the authors could include a section explaining the Hondius outbreak.

2. Related to the first point, in lines 64-66, the authors dive right into discussing the “likelihood that the M/V Hondius ANDV cluster could progress into a sustained epidemic”. Before this section, there is no context of what the N/V Hondius ANDV cluster is.

3. In lines 69-70, the authors mention that the sequences derived from the ANDV cluster have two synonymous mutations with respect to ANDV reference sequences from Argentina (1997 and 2018) and cite the Virological.org post by Dr. Gustavo Palacios. However, that is not what the post mentions; instead, the post describes two nonsynonymous mutations arising in the L segment between the first three sequences generated in South Africa, and they argue that this is a sign of a single spillover event as the origin of the index case. The original publication then mentions «98.76% and 98.75% based on the L segment, 98.71% and 98.68% on the M segment and 98.73% and 98.73% on the S segment», these are with respect to the reference sequences from 1997 and 2018, which is more than two SNPs. Therefore, the authors should rectify the assertion of only two SNPs in the Hondius sequences, it would be evolutionarily impossible for an RNA virus to only accumulate two mutations in almost 30 years.

Authors’ response:

Thank you for reviewing our manuscript and providing us with useful feedback. We have addressed your comments accordingly.

We have added a paragraph introducing the *M/V Hondius* ANDV outbreak for context on Lines 60-66 and highlighted the changes using Track Changes tools and in yellow. The following sentence was deleted as the M/V Hondius ANDV cluster has been declared over: “Additional cases associated with the M/V Hondius cluster may still be identified within the recognised incubation period”

Refer to abovementioned response addressing this comment. We have added, on lines 60-66, context of what the *M/V Hondius* ANDV cluster is. We have also added additional text to improve clarity on lines 71-73.

We thank the reviewer for identifying this ambiguity in Lines 69-70. We agree that our original wording incorrectly conflated the limited sequence variation observed among cases within the 2026 *M/V Hondius* cluster with the genetic divergence between the outbreak sequences and historical ANDV sequences from Argentina. The preliminary genomic analysis reported that the S and M consensus sequences generated from the cluster were identical to each other, whereas two SNPs were detected among the L-segment sequences (C2139T in case 5 and G576A in case 7). Importantly, the report describes both of these SNPs as synonymous. In contrast, comparison with ANDV sequences from Argentina in 1997 and 2018 showed approximately 98.7% nucleotide identity across the three genome segments, indicating considerably greater divergence from these historical reference sequences. We have therefore revised the manuscript to clearly distinguish within-cluster genomic variation from divergence relative to historical ANDV strains and have removed the incorrect implication that the 2026 outbreak virus differed from the 1997 and 2018 strains by only two substitutions. Thus, we adjusted and replaced the text to improve clarity on lines 79-90, as indicated in the Track Changes and highlighted in yellow.

# Reviewer report: REVIEWERS’ COMMENTS

## REVIEWER #1

The authors have satisfactorily responded to all comments and questions raised, and the revised manuscript is suitable for publication.

## REVIEWER #2

I would like to thank the authors for thoroughly implementing the requested changes. The manuscript is clear, informative, and has my recommendation for acceptance.
